# Modulation of the gut microbiome with nisin

**DOI:** 10.1038/s41598-023-34586-x

**Published:** 2023-05-16

**Authors:** Catherine O’Reilly, Ghjuvan M. Grimaud, Mairéad Coakley, Paula M. O’Connor, Harsh Mathur, Veronica L. Peterson, Ciara M. O’Donovan, Peadar G. Lawlor, Paul D. Cotter, Catherine Stanton, Mary C. Rea, Colin Hill, R. Paul Ross

**Affiliations:** 1 APC Microbiome Ireland, University College Cork, Co. Cork, Ireland; 2 Food Biosciences Department, Teagasc Food Research Centre, Moorepark, Fermoy, Co. Cork, Ireland; 3 Microbiology Department, University College Cork, Co. Cork, Ireland; 4 Pig Development Department, Teagasc Animal & Grassland Research & Innovation Centre, Moorepark, Fermoy, Co. Cork, Ireland

**Keywords:** Antimicrobials, Metagenomics, Microbial communities, Microbiome

## Abstract

Nisin is a broad spectrum bacteriocin used extensively as a food preservative that was identified in *Lactococcus lactis* nearly a century ago. We show that orally-ingested nisin survives transit through the porcine gastrointestinal tract intact (as evidenced by activity and molecular weight determination) where it impacts both the composition and functioning of the microbiota. Specifically, nisin treatment caused a reversible decrease in Gram positive bacteria, resulting in a reshaping of the Firmicutes and a corresponding relative increase in Gram negative Proteobacteria. These changes were mirrored by the modification in relative abundance of pathways involved in acetate, butyrate (decreased) and propionate (increased) synthesis which correlated with overall reductions in short chain fatty acid levels in stool. These reversible changes that occur as a result of nisin ingestion demonstrate the potential of bacteriocins like nisin to shape mammalian microbiomes and impact on the functionality of the community.

Bacteriocins are antimicrobial peptides produced by many bacterial species^1^. Nisin is a bacteriocin produced by *Lactococcus lactis* that has a broad spectrum of activity against Gram-positive bacteria^2,3^. Nisin A has been approved for use as a food preservative by the US Food and Drug Administration (FDA) (US Food and Drug Administration, 1988) and the European Food Safety Authority (EFSA; E number E234) and is therefore consumed by humans^4^. Nisin (in the form of the commercially available Nisaplin) has been fed to rats without adverse effects, at doses of up to 239 mg per kg body weight^5^. Nisin has in vitro efficacy against Gram positive gut pathogens such as *Clostridioides difficile*^6^ and, in combination with cinnamaldehyde and ethylene diamine tetra-acetic acid (EDTA), has been shown to control the growth of Gram negative enterotoxigenic *Escherichia coli* (ETEC)^7^. Nisin has also been shown to have in vivo efficacy on the murine^8,9^ and chicken microbiomes^10^, as well as *ex-vivo* efficacy on the human microbiome^11^. Nonetheless, no studies have shown its in vivo efficacy on large mammals so far. Because of its proteinaceous nature, it has been assumed that nisin will be broken down in the upper intestine due to exposure to proteolytic enzymes, as we have shown previously for other bacteriocins, namely lacticin 3147^12^ and thuricin CD^13^. If nisin does not reach the lower GIT intact^14,15^ it should not affect the gut microbiota when ingested orally. In this study, we fed high concentration nisin to post-weaning piglets to determine its impact on the gut microbiota using 16S sequencing, metagenomics (shotgun), GC–MS and Maldi-Tof MS. We also included an ethyl cellulose-based preparation to protect (encapsulate) nisin against possible degradation in the upper GIT^16^ and compare it with non-encapsulated nisin, something that was not tested in previous studies.

Here, we demonstrate that intact non-encapsulated nisin is delivered to the lower GIT of pigs and causes significant but reversible changes in microbial composition, short-chain fatty acid (SCFA) levels and in specific metabolic pathways throughout the treatment period.

## Results

**Figure 1:**
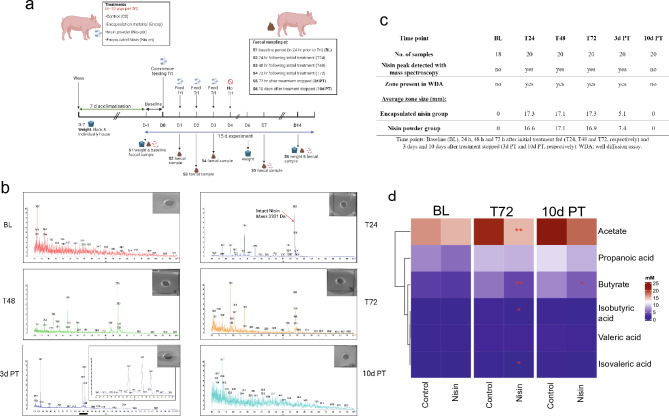
Overview of the experiment, mass spectroscopy and metabolomics results. (**a**) Overview of the treatment groups and the sampling timeline. (**b**) MALDI TOF Mass spectrophotometry analysis and activity assays to detect nisin (pictures). Detection of intact nisin (red arrows) in the faeces of pig in the nisin powder treated group at Baseline (BL), 24 h following initial treatment (T24), 48 h following initial treatment (T48), 72 h following initial treatment (T72), 72 h after initial treatment stopped (3d PT) and 10 days after initial treatment stopped (10d PT). Smaller zones and Nisin masses were found in Day 7 and no zones or masses found on day 14 (1 in 10 dil). (**c**) Biological activity of nisin and nisin peak detection in porcine faecal samples, using Maldi TOF mass spectrophotometry. (**d**) SCFA concentration (mM) in control (here Ctl and Encap grouped together) versus nisin groups (here nis-en and nis-pdr grouped together) over BL, T72 and 10d PT (Wilcoxon Rank Sum test, *p < 0.05, **p < 0.01, ***p < 0.001).

The aim of this study was to evaluate if orally ingested nisin could reach the gut intact and modulate the gut microbiome of pigs (Fig. 1a). Due to the proteinaceous nature of nisin we used both non-encapsulated (Nis-pdr) and encapsulated nisin (Nis-en), which were directly added to the pig feed. This study also contained a no treatment control group (Ctl) and a group that was fed the material that was used to encapsulate the nisin (Encap). Finally, we monitored the composition and functionality of the gut microbiota following cessation of treatment, to determine how long any changes persisted.

### Detection of nisin in the faeces

Both MALDI TOF mass spectrometry and activity assays confirmed the presence of intact nisin and antibacterial activity in the faeces of every pig in both the encapsulated nisin and nisin powder treated groups throughout the treatment period (Fig. 1b). This analysis revealed a peak corresponding to the molecular mass of intact nisin (3331.05 Da) on the treatment days in the two nisin-treated groups (Nis-en and Nis-pdr) (see supplementary Fig. 5 for Nis-en). Bioactivity, measured by well diffusion assays, was confirmed in the faeces of all pigs in the encapsulated nisin and nisin powder groups for three days after cessation of treatment. However, the zone sizes were smaller than those observed on the nisin treatment days (Fig. 1b,c). Intact and active nisin was not detected in any of the baseline (BL) samples, the encapsulant group (Encap) or in the no treatment control group (Ctl), or in any of the samples taken ten days after treatment ceased (10d PT).

### Short-chain fatty acid concentrations decreased in nisin treated pigs during treatment

Faecal samples taken at the baseline (BL), after four consecutive days of treatment (T72) and ten days after treatment ceased (10d PT) were analysed to determine the SCFA concentration. A targeted method was used to analyse the samples for 10 different compounds (acetate, formic acid, propionic acid, butyrate, isobutyric acid, isovaleric acid, valeric acid, 4-methylpentanoic acid, hexanoic acid and heptanoic acid). 4-methylpentanoic acid was only found in five samples, and in all cases at levels very close to the limit of detection. Hexanoic acid and heptanoic acid were excluded from further analysis because the levels in the baseline (BL) samples were below the limit of detection for 50% or more of samples. Hence, six compounds were included in the statistical analysis: butyrate, acetate, isovaleric acid, isobutyric acid, valeric acid and propionic acid (supplementary Table 2). To test for differences related to the treatment of the subjects, Wilcoxon Rank Sum test were performed comparing control (Ctl and Encap grouped together) and treatment (nis-en and nis-pdr grouped together) groups at the different time points. Results showed a significant decrease in the level of acetate (*p* = 0.004, difference of 40.13%), butyrate (*p* = 0.0012, difference of 66.15%), isobutyric acid (*p* = 0.03, difference of 36.47%) and isovaleric acid (*p* = 0.035, difference of 36.36%) in the nisin treated group compared to the control group at T72 (Fig. 1d). No other significant differences were observed across treatment groups. Ten days after cessation of treatment, only the butyrate level remains significantly lower (*p* = 0.048). The highest variation in SCFA levels across samples was observed 10 days post-treatment.

### Growth performance of pigs did not change in response to treatment

Feed conversion ratio (FCR) is a common index used to measure feed efficiency in pigs. Average daily weight gain and average daily feed intake increased across the trial period. There was no difference in pig live weight between the treatment groups (supplementary Fig. S1a). This was not unexpected given the short duration of the study. The growth performance of the pigs is shown in supplementary Fig. S1b.

### Nisin Z treatment reversibly affected the gut microbiota composition

**Figure 2:**
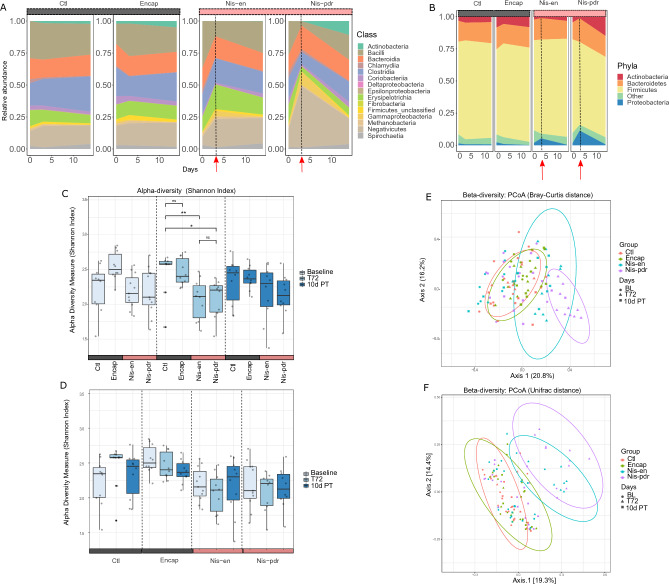
Microbiome community composition and beta diversity are significantly different in nisin treatment at T72, while alpha-diversity is not. (**a**) Sankey plot showing the relative abundance of the microbiome in the different control versus nisin treatment groups at the Class level. The red arrows and the dotted line correspond to the highest dose of nisin in the samples. (**b**) Sankey plot showing the relative abundance of the microbiome in the different control versus nisin treatment groups at the Phylum level. The red arrows and the dotted line correspond to the highest dose of nisin in the samples. (**c**) Alpha diversity, measured using the Shannon index, changing significantly between groups at T72 (Wilcoxon Rank Sum test, *p  < 0.05, **p < 0.01). The grey and pink area denote the control groups (Encap and Ctl) and nisin treatment groups (Nis-en and Nis-pdr), respectively. (**d**) Alpha diversity, measured using the Shannon index, shown within each group. No significant differences were observed. (**e**) PCoA ordination (Bray–Curtis distance) showing that samples from Nis-en and Nis-pdr at T72 mostly group together (blue and pink ellipses, respectively). Ellipses represent a 95% CI around the cluster centroid for each study area group at T72. (**f**) PCoA ordination (Unifrac distance) showing that samples from Nis-en and Nis-pdr at T72 mostly group together (blue and pink ellipses, respectively). Ellipses represent a 95% CI around the cluster centroid for each study area group at T72.

We assessed the microbial community composition of the different treatment groups at three different time points (BL, T72, 10d PT) using both shotgun metagenomics and 16S rRNA profiling. Firmicutes was the most abundant phylum across all treatment groups in the baseline samples (mainly *Bacillus*, *Clostridium* species, Erysipelotrichia and Negativicutes), followed by the Bacteroidetes, whereas Actinobacteria and Proteobacteria were less abundant (Fig. 2a,b). Throughout the study, changes in the microbiota composition across treatment groups were observed. After three consecutive days of treatment (T72), there were significant differences in the overall community composition between untreated and treatment groups (*p* < 0.001, PERMANOVA). In particular, there was a reshaping of the Firmicutes family composition in the nisin treated groups, with an increase of Negativicutes and a decrease of *Bacillus* species. There was also an observed increase in Proteobacteria (mainly Gammaproteobacteria) in the nisin treated groups, with the Nis-pdr group having the largest increase (360% relative to BL). This observed change in the nisin treated groups reverted to a composition similar to baseline levels ten days after the treatments ceased. The microbiota composition in the control and encapsulant groups remained relatively stable across the trial period, with some notable continuous increase in Actinobacteria over time, as well as small changes in the Firmicutes (increase of *Bacillus* species accompanied to a decrease in *Clostridium* species) at T72 in the encapsulant group. Overall, we observed the same results with the 16S rRNA (supplementary Fig. S2).

Alpha diversity, measured using the Shannon index, shows that at baseline there are no significant differences between the treatment groups (Fig. 2c). There are significant differences at the end of the treatment period (T72) between nisin treated groups compared to the no treatment control group (Wilcoxon Rank Sum test, *p* < 0.05 and *p* < 0.01 for the Nis-en and Nis-pdr group, respectively). However, this difference was not observed within each group when comparing T72 with baseline and 10d PT, suggesting that nisin treatment did not have a significant impact on alpha-diversity (Fig. 2d). No significant differences were observed between the control and encapsulant groups following three consecutive days of treatment.

To further investigate the diversity differences of the treatment groups and time points, beta diversity based on Bray–Curtis (Fig. 2e) and Unifrac (Fig. 2f) distances were assessed. The baseline samples of all treatment groups clustered together. Following three consecutive days (T72) of feeding the treatments, there was a shift in diversity in the nisin treated groups compared to the non-nisin treated groups (BH adj. *p* < 0.01, PERMANOVA of Bray–Curtis and Unifrac distances). Ten days after feeding of the treatments had ceased the beta diversity in the nisin treated groups was again comparable to that of the non-nisin treated groups.

### Gram-positive bacteria were reversibly decreased while Gram-negative bacteria were reversibly increased in nisin treated groups during treatment

**Figure 3:**
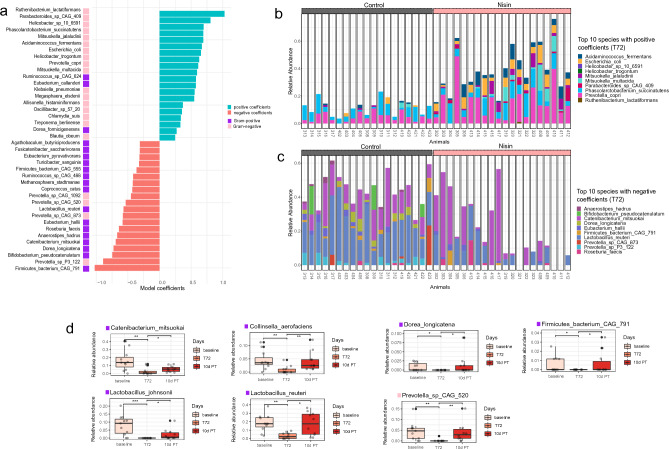
Gram-positive bacteria are significantly decreased during nisin treatment while gram-negative bacteria significantly increase. (**a**) Model coefficients of the multinomial regression analysis made with Songbird (model: species ~ nisin treatment x days) ranked according to nis-pdr at T72. Species with high coefficients (positive and more abundant in nis-pdr T72, blue, or negative and less abundant in nis-pdr T72, red; pink, gram-positive, purple, gram-negative) were best able to distinguish nis-pdr group during treatment at T72. (**b**) Community composition at the individual level of the top 10 species with positive coefficients (grey, control groups, pink, nisin treatment). (**c**) Community composition at the individual level of the top 10 species with negative coefficients (grey, control groups, pink, nisin treatment). (**d**) Boxplot showing the relative abundance of the species that were significantly decreased during nis-pdr T72 only compared to both nis-pdr baseline and nis-pdr 10d PT (Wilcoxon Rank Sum test, *p < 0.05, **p < 0.01, ***p < 0.001). All are gram-positive (purple) except Prevotella sp CAG 520 (pink).

To identify which species changed the most relative to each other in the nisin powder treated group after three consecutive days, a differential abundance analysis using the Differential Ranking (DR) method was used based on relative abundances. Differential Ranking reformulates the differential abundance at the species level as a multinomial regression based on log fold change. Species with high coefficients (Fig. 3a) (positive (blue) or negative (red); reference for comparison are nisin treatments groups at T72) were best able to distinguish species in the nisin treatment group during the treatment period at T72.

Of the 20 bacterial species that were differentially decreased in the nisin treatment groups at T72, sixteen were Gram-positive (Fig. 3a,c). In particular, *Anaerostipes hadrus*, *Bifidobacterium pseudocatenulatum* and *Dorea longicatena* were undetectable during nisin treatment in all the pigs and are thus most responsive to nisin. The Gram-negative bacteria that significantly decreased in the treatment group at T72 were all from the *Prevotella* genus. Other Gram-positive species such as *Catenibacterium mitsuokai, Collinsella aerofaciens*, *Lactobacillus johnsonii* and *Lactobacillus reuteri* were more significantly or only decreased in Nis-pdr at T72 (Fig. 3c,d). Despite considerable variations at the individual level, Nis-pdr treatment explained the between-sample variability. A focus on Nis-pdr revealed that the species that were significantly decreased at T72 all reverted to pre-treatment levels after ten days (Fig. 3d).

Of the twenty bacterial species that were differentially increased in the nisin treatment groups at T72, 17 were Gram-negative (Fig. 3a,b). Species such as *Acidaminococcus fermentans*¸ *Esherichia coli*, *Phascolarctobacterium succinatutens* and *Prevotella copri* were differentially increased in nearly all the nisin treated individuals at T72. The only Gram-positive species that were differentially more abundant in nisin treated individuals at T72 were *Dorea formicigenerans*, *Eubacterium callanderi*and *Ruminococcus* sp. CAG 624.

Taken together the results show that Gram-positive bacteria were under-represented in the nisin treated groups while Gram-negative bacteria were over-represented, especially in the Nis-pdr group on the days nisin was fed. However, following cessation of treatment these microbial communities in the nisin treated group returned to levels similar to pre-treatment levels as seen 10 d PT.

### Nisin treatment impacts the functional profile of the gut microbiome

**Figure 4:**
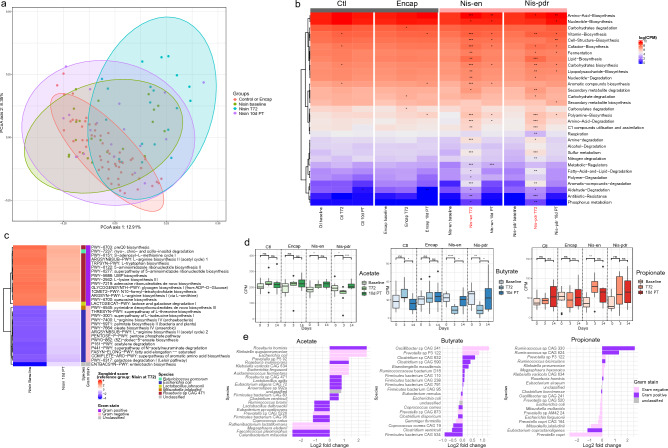
Functional analysis of the control versus treatment groups. (**a**) PCoA ordination (Bray–Curtis distance) of the HUMAnN gene counts (counts per million) for the Nis-en and Nis-pdr at T72 versus the other groups. (**b**) Heatmap showing the functional composition (log(HUMAnN count per million)) at the highest MetaCyc pathway level hierarchy of the different groups at the different time points (grey area, control groups, pink area, treatment groups; the red arrows correspond to the highest dose of nisin in the samples). The pathways are hierarchically clustered using Euclidean distance. (**c**) Heatmap showing the top 30 most differentially abundant HUManN pathways according to their Songbird scores (model: pathways ~ nisin treatment x days) for the groups Nisin (Nis-en and Nis-pdr) at Baseline versus T72 (left) and 10d PT versus T72 (right). These pathways are stratified by species (shown on the right bar). We show here the top 15 most positive (i.e., more associated with Nisin at Baseline (left) or 10d PT (right)) and the top 15 most negative pathways (i.e., more associated with Nisin at T72). (**d**) Abundance (HUMAnN count per million) of the pathways involved in SCFA production in the different groups at different time points and their differences (Wilcoxon Rank Sum test, *****p < 0.0001, ***p < 0.001, **p < 0.01, *p < 0.05, ns: non-significative). (**e**) Log2 fold changes of the top 20 species with highest and lowest gene abundance log2fold changes in nisin treated groups relative to their respective baseline.

The functional profile of the microbial community was assessed using shotgun metagenomics. Principal Coordinate Analysis (PCoA) of gene counts (counts per million) revealed that the microbial functional profile in nisin treated groups at T72 was significantly different from the other groups (PERMANOVA, BH adj. *p* < 0.01) (Fig. 4a). Moreover, the nisin treated groups exhibited a divergent metabolic pathway profile during the treatment period at T72 for most of the pathways found in the metagenomics data (MetaCYC pathways, Wilcoxon Rank Sum test, *p* < 0.01) (Fig. 4b). Pathways such as Amino − Acid − Biosynthesis and Nucleotide—Biosynthesis did not change across the treatment groups and time points. These pathways are used by all bacteria, and we expect that changes in bacterial composition do not affect them. On the contrary, the relative abundance of pathways such as Vitamin Biosynthesis, Lipid Biosynthesis, Sulfur Metabolism, Fatty-Acid-and-Lipid-Degradation, Aromatic-compounds-degradation all increased in the nisin treated groups at T72 while the Respiration pathway decreased (*p* < 0.01 based on Wilcoxon Rank Sum test of log2 fold change with BL as reference). Some pathways, such as the Cell-Structure-Biosynthesis pathway, are probably directly influenced by nisin due to its action on cell membranes. This difference in pathway abundances also reflects the change in bacterial composition following nisin treatment. To investigate this further, we looked at the most differentially abundant metabolic pathways at a lower hierarchy, stratified by species, using the previously described tool Songbird (Fig. 4c). Nisin treated groups showed similar differential abundance profiles at both baseline and 10d PT. Pathways more associated with nisin treated groups at T72 (in blue in Fig. 4c) belong to Gram-negative bacteria previously found to be more abundant in these groups at these time points (i.e., *Escherichia coli* and *Mitsuokella jalaludinii*). Similarly, pathways that were more associated with nisin treated groups at baseline and 10d PT (and thus mostly absent at T72) belong to Gram-positive bacteria previously identified as more abundant in these groups and at these time points (i.e., *Lactobacillus johnsonii* and *Roseburia* sp CAG 471), except for *Butyricicoccus porcorum* which was not identified previously. Of note, two pathways (L-arginine biosynthesis and galactose degradation) were both associated with nisin treated groups at T72 and nisin treated groups at baseline and 10d PT, but are carried by different bacteria, probably reflecting the replacement of Gram-positive species by Gram-negative species performing, at least in part, similar functions. Some functions seem, nonetheless, to be unique to the nisin treated groups at T72. For example, the pathway that was the most associated with nisin treated groups at T72 was enterobactin biosynthesis, a siderophore primarily found in Gram-negative bacteria.

We then assessed the representation of genes involved in SCFA production in the different groups at different time points. There was a significant decrease in the abundance of genes involved in production of butyrate in the encapsulated nisin group and the nisin powder treated group between the baseline and T72 (Wilcoxon Rank Sum test, *p* < 0.0001 and *p* < 0.01 respectively) (Fig. 4d). At 10 d PT, there was a significant increase in the representation of these genes (Wilcoxon Rank Sum test, *p* < 0.05). Similarly, this pattern was observed in the genes associated with acetate production. A significant increase in the genes involved in propionate production between the baseline and T72 in Nis-en was observed. Following cessation of treatment, these levels returned to baseline levels. We also looked at the abundance of genes involved in SCFA production stratified by the species carrying them (Fig. 4e). The top 10 species with the highest and lowest log2 fold change of gene abundance in nisin treatment related to BL showed that the species responsible for SCFA production changed from mostly Gram-positive to mostly Gram-negative for acetate and butyrate during nisin treatment. In particular, Firmicutes are replaced by Gram-negative bacteria such as *E.coli* for acetate or *Prevotella* sp. for butyrate. Surprisingly, the opposite was observed for propionate, with an increase of gene abundance carried by Gram-positive species during nisin treatment, namely *Ruminococcus* sp. This might be related to the increase of some *Ruminoccocus* species during nisin treatment shown previously (Fig. 4a).Overall, these results show a functionally altered community structure during nisin treatment mainly due to the replacement of Gram-positive bacteria by Gram-negative bacteria, which is restored after feeding of the treatment has ceased.

## Discussion

We reveal that nisin, used at a concentration well below the no-observed-adverse-effect level (NOAEL)^17^, remained intact during gut transit and successfully modified the porcine gut microbiota, reducing the relative abundance of many Gram-positive microorganisms. We hypothesise that the post-translational modifications present in lantibiotics such as nisin protect these bacteriocins in the gut environment. In contrast, pre-nisin peptides synthesized without these modifications are readily digested by trypsin and chymotrypsin (supplementary Fig. S4). The findings from our study challenge a major dogma in bacteriocin research—that bacteriocins are broken down in the gut by proteases rendering them inactive and hence unsuitable for oral delivery unless encapsulated. On the contrary, we demonstrate nisin can survive gastric transit in sufficient amounts to modify the gut microbiome when ingested by a large animal and highlights the potential to use lantibiotics as a tool to modify the gut microbiome.

Gut microbiota composition was impacted in the nisin treated groups during treatment but returned to levels similar to the untreated groups as soon as three days after treatment ceased, while diversity was not significantly impacted. We additionally observed that nisin powder was more efficient than encapsulated nisin. Treatment with nisin resulted in the depletion of Gram-positive bacteria such as *Lactobacillus* and an increase in the relative abundance of Gram-negative bacteria such as *Escherichia coli* during treatment*.* The nisin treatment has a selective effect on Gram-positive bacteria, and these bacteria are possibly replaced by Gram-negative species. Interestingly, some Gram-negative bacteria, especially *Prevotella*, are sensitive to nisin treatment, as shown previously with nisin Z^18^, while some Gram-positive bacteria, for example *Ruminococcus,* appear to be immune to nisin. Additionally, high levels of *Catenibacterium* were found in both the encapsulant control group and encapsulated nisin group, compared to the control and nisin powder groups. This suggests that the *Catenibacterium* are potentially degrading the cellulose encapsulant material and using it as an energy source.

We also observed changes at the functional level in nisin treated groups during nisin treatment, with the most differentially increased pathways all related to Gram-negative bacteria and the most differentially decreased pathways all related to Gram-positive bacteria, reflecting the changes in taxonomic composition. Some pathways from Gram-positive species (e.g., *Roseburia*) were replaced by the same pathways present in Gram negative species (e.g. *E. coli*), pointing to potential ecological niche replacements. However, other pathways unique to Gram-negative bacteria also increased (e.g., enterobactin associated with *E. coli*), showing that replacement of Gram-positive by Gram-negative bacteria was not completely functionally equivalent, something that is also seen in the higher order hierarchy pathways that were almost all different from BL (Fig. 4b). Additionally, we observed the increase in pathways potentially related to adaptations by both Gram-positive and Gram-negative bacteria to nisin-induced stress. For example, we observed an increase in pathways associated with vitamins (especially menaquinone, see supplementary Fig. S3). In many bacteria, the synthesis of menaquinone, lipid-soluble molecules that insert themselves into the bacterial cell membrane, may be affected by the state of the cell membrane^19^, providing insight into the impact nisin can have on the microbiota dependant on its mechanism of action that involves binding to lipid II.

We observed an overall decrease in SCFA levels in the nisin treated groups at T72. These recovered ten days after treatment had ceased in the nisin treated groups, albeit returning to levels lower than the group fed the encapsulant material or the no treatment control group. *Lactobacillus* and *Catenibacterium* have been shown to metabolize carbohydrates including oligosaccharides and starch, which are fermented in the large intestine to SCFAs and can then be utilized by the pigs^20–23^. Their decrease probably explains, at least in part, the decrease in SCFA. This is especially the case with the decrease of *Catenibacterium* for acetate, which is not fully compensated by Gram-negative species such as *E. coli* (Fig. 4e). For butyrate, Gram-positive Firmicutes are replaced by *Oscillibacter* and *Prevotella*, but as other Gram-positive bacteria still continue to play an important role in butyrate production while decreasing in relative abundance, the overall concentration of butyrate decreases. Interestingly, for propionate, Gram-negative bacteria seem to be replaced by the Gram-positive bacteria *Ruminococcus* which seems to be resistant to nisin. By affecting luminal SCFAs, intestinal microbiota can modulate intestinal incretin excretion, and hence, exert another influence on glucose regulation^24–26^.

This study highlights the potential of nisin to modulate the microbiome when taken orally. The temporary effect on the microbiome was to remodel Gram positive and negative ratios and in so doing reduce SCFA production. Although nisin A used in this study is broad spectrum we have been able to change the specificity of the lantibiotic via peptide-engineering approaches^27,28^ Added to this, the rapidly expanding number of Type I post-translationally modified bacteriocins with differing specificities opens up opportunities to use them for microbiome editing applications (supplementary Fig. S4). Moreover, since nisin kills a range of undesirable bacteria including pathogenic streptococci and clostridia it could be used as an alternative to antibiotics in some instances. An additional benefit of nisin as highlighted in this study is the ability of the microbiota to return to pre-treatment composition compared with the longer lasting impact of some antibiotics treatment which can persist for up to two years post-treatment^29^.

## Materials and methods

### Dietary treatments

There were four dietary treatment groups as follows: (i) a standard non-medicated link diet (control group; Ctl); (ii) the link diet supplemented with 150 mg/kg body weight of nisin powder (Nis-pdr) (Handary, Brussels, Belgium; high content powder nisin ZP (95% content/ultrapure; % weight/weight; hydrous potency ≥ 38,000 IU/mg)); (iii) the link diet supplemented with 850 mg/kg body weight of encapsulated nisin ZP (Nis-en) (Sublimity Therapeutics, Dublin, Ireland); and (iv) the link diet supplemented with 110 mg/kg body weight of the encapsulant material (Encap). The encapsulant material was ethyl cellulose (98%) (DOW Chemical Company Limited, Staines, England; gifted by Sublimity Therapeutics). The dose of nisin administered in the encapsulated nisin (Treatment 3) was equivalent to that of the nisin powder treated group (Treatment 2). The amount of encapsulant administered in Treatment 4 was equivalent to the amount in the encapsulated nisin formulation in Treatment 3. Treatments in powder form were top-dressed to a small amount of feed with 5–10 ml water in the morning to ensure that all the treatments were consumed each day.

### Animal management

This study was conducted in the Pig Development Department, Teagasc, Moorepark, Fermoy, Cork, Ireland. A schematic depicting the trial structure is presented in Fig. 1. Piglets (Large White X Landrace) were weaned at 28 (± 3) days, housed in groups of intact litters, and fed a starter diet for 6 days with each pig ear tagged with a unique number for identification purposes. From this group of 150 pigs, 40 males were selected and blocked on body weight and litter origin, and then randomly assigned to one of the four dietary treatments detailed above (N = 10 pigs/treatment). The pigs were individually housed in fully slatted pens (1.2 m × 0.9 m) and were each provided with a non-medicated link diet for a seven-day acclimatisation period. The room temperature was maintained at 28–30 °C during the first week post-weaning and then reduced by 2 °C each week until a temperature of 22 °C was achieved in the room. Feed and water were provided on an ad libitum basis, lighting was ensured for ≥ 8 h/day and environmental enrichment was provided. Following the seven-day acclimatisation period, dietary treatment commenced. Dietary treatment was provided for four days, following which pigs were again fed the common un-medicated link diet for ten days until the end of the trial. Proximate analysis and amino acid composition of the diets used are presented in Supplementary Table 1. All pigs were individually weighed on Day 0 (D0) (Fig. 1) prior to the start of the trial (baseline (BL)). Growth performance was assessed by weighing all pigs on two further occasions (i) mid-way through the trial (Day 6; two days after treatment had ceased) and (ii) at the end of the trial (Day 14; ten days after treatment had ceased). Voluntary feed intake was recorded daily between the start and end of the trial, and this divided by the average daily gain was used to calculate feed conversion ratio (FCR).

### Treatment groups, sampling time points and sample collection

Faecal samples were taken before the treatment commenced to determine the baseline (D0; BL) microbiota. Further faecal samples were taken 24 h after the initial treatment (T24) was fed, 48 h after initial treatment was fed (T48) and 72 h after initial treatment was fed (T72). These samples were taken to assess the immediate impact of treatment on the porcine gut microbiota. Faecal samples were also taken towards the end of the trial, three days after treatment ceased (3d PT) and ten days after treatment ceased (10d PT). These samples were taken to observe the recovery of the gut microbiota when treatment ceased. The fate of the ingested nisin was also determined from these samples. Faecal samples were collected freshly voided, when available; otherwise, they were collected following rectal stimulation. Samples were collected into sterile pots and stored on ice briefly (< 2 h) and sub-sampled into sterile 2 ml Eppendorf tubes. Samples were then immediately snap-frozen in liquid nitrogen and stored at − 80 °C for DNA extraction and SCFA analysis or at − 20 °C for screening for the presence and activity of nisin (for well diffusion assays (WDAs) and MALDI TOF mass spectrometry (MALDI TOF MS)).

### DNA extraction

DNA was extracted from 200 mg faeces from each pig at BL, T24, T48, T72, 3d PT and 10d PT using the Zymo Research ZR faecal DNA kit (Cambridge Biosciences, Cambridge, United Kingdom), according to the manufacturer’s specifications.

### 16S rRNA library preparation

DNA was extracted from the faecal samples taken at BL, T24, T48, T72, 3d PT and 10d PT. 16S rRNA gene amplicons (V3–V4 region) were generated and sequenced using the Illumina MiSeq™ platform. The V3-V4 variable region of the 16S rRNA gene was amplified from 240 faecal DNA samples using the 16S metagenomic sequencing library protocol (Illumina San Diego, CA). Samples were quantified and libraries were prepared for sequencing as described previously^35^. In brief, the 16S rRNA amplicon sequencing libraries were prepared as follows: two PCR reactions were completed on the template DNA. DNA was firstly amplified with primers specific to the V3–V4 region of the 16S rRNA gene, which also incorporate the Illumina overhang adaptor (forward primer 5′TCGT CGGC AGCG TCAG ATGT GTATAAGA GACA GCCT ACGG GNGG CWGCAG; reverse primer 5′GTCT CGTG GGCTCGGA GATG TGTA TAAG AGAC AGGA CTAC HVGG GTAT CTAATCC). Following this, a second PCR reaction was performed with two indexing primers (Illumina Nextera XT indexing primers) added to allow for demultiplexing. Samples were analysed on the Illumina MiSeq at the Teagasc Sequencing Centre, Moorepark, Fermoy, Co. Cork, Ireland using the Miseq 600 cycle v2 kit and following standard Illumina sequencing protocols. Following sequencing, the data were analysed to establish the effect of nisin treatment on the composition of the gut microbiota. The average number of OTUs per sample was 123,814 +/− 34,649 with a range between 24,578 and 231,778.

### Shotgun metagenomic library preparation

Paired-end sequencing libraries were prepared from the extracted DNA from 120 samples (BL, T72 and 10d PT) using the Illumina Nextera XT library preparation kit (Illumina) followed by sequencing on the Illumina NextSeq 500 platform using high-output chemistry (2 × 150 bp) according to the manufacturer’s instructions at the Teagasc Sequencing Centre.

### Matrix-assisted laser desorption/ionization time of flight mass spectrometry (MALDI TOF MS)

Nisin was extracted from the faecal samples as follows: samples were suspended in 1 mL of 70% isopropyl alcohol containing 0.1% trifluoroacetic acid (TFA) and (IPA), vortexed thoroughly and allowed to stand at room temperature for 30 min and centrifuged for 5 min at 16,000 g and the supernatant retained. The centrifugation step was repeated a further three times with the supernatant being retained each time. The molecular mass corresponding to the nisin peak was observed using MALDI TOF MS with an Axima TOF2 (Shimadzu Biotech, Kyoto, Japan), as previously described^25^.

### Faecal water preparation and SCFA analysis

Four hundred milligrams of frozen faecal material was placed in a sterile 2 ml microcentrifuge tube. Eight hundred microlitres of sterile water was added to the 2 ml microcentrifuge tube. The sample was vortexed vigorously for 30 s to generate a faecal slurry. Samples were then centrifuged at 12,000 g for 30 min at 4 °C. The supernatant was removed and transferred to a new 2 ml microcentrifuge tube. This step was repeated. The supernatant was then centrifuged again at 12,000 g for 30 min at 4 °C. This supernatant was transferred to a 0.2 µM costar spin centrifuge tube (Sigma-Aldrich, UK) and filtered by centrifugation at 12,000 g for 10 min at 4 °C. The filter was removed from the tube and the faecal water was stored at − 20 °C prior to metabolomic analysis. Samples were analysed by MS-Omics ApS, Frederiksberg, Denmark to determine metabolite levels, including SCFAs.

### Antibacterial activity assay

Antibacterial activity in the porcine faecal samples was estimated using well diffusion assays (WDAs)^30^ in agar plates seeded with *Lactobacillus delbrueckii* ssp *bulgaricus* LMG6901, as detailed previously^31^. Faecal water, prepared as above, was used in the assays. The samples were dispensed into the wells of the seeded agar in 50 μL aliquots and the agar plates were incubated anaerobically overnight at 37 °C. Antibacterial activity resulted in zones of inhibition around the wells. Samples from animals that had not consumed nisin were used as negative controls to ensure that zones of inhibition were due to nisin inhibition.

### Processing of 16S Illumina sequencing

Paired-end reads were trimmed and quality filtered using PRINSEQ version 0.20.4^32^. Resulting sequences had a mean quality score greater than 20. Forward and reverse paired-end sequences were then joined with a minimum overlap of 10 bp using fastq-join^33^. Sequences were clustered into Operational Taxonomic Units (OTU) using closed-reference algorithm in USEARCH version 7.0^34^ and chimeras were removed with the RDP Gold reference database.

### Metagenomic reads quality control, taxonomic and metabolic functional annotation

The metagenomic shotgun sequencing reads were qualified and trimmed, and human reads (hg19 human reference genome) were filtered with KneadData v0.10.0 with the default options. Qualified sequencing reads were then taxonomically profiled at the species level by MetaPhlAn v3.0.1^35^ and the other options with default settings. MetaPhlAn depends on a set of unique clade-specific marker genes (~ 1.1 million) identified from ~ 100,000 reference genomes (~ 99,500 bacterial and archaeal and ~ 500 eukaryotic genomes) to provide pan microbial quantification at the species level, including bacteria, archaea, microbial eukaryotes, and viruses^35,36^. Functional annotations including MetaCyc pathways and gene-family abundances were achieved by HUMAnN v3.0.0^35,37^. Gene family files were regrouped with “humann_regroup_table” function from HUMAnN to “go_uniref90” (Gene Ontology), “level4ec_uniref90” (Enzyme Commission), “ko_uniref90” (KEGG Orthology) and HUMAnN pathways (count per million) and HUMAnN genes (count per million) terms, respectively.

### Microbial community composition and group comparison

The taxonomic matrix obtained with MetaPhlAn v3.0.1 was used for Alpha-diversity (Shannon index) and Bray–Curtis beta diversity analysis using the R package “PhyloSeq” v1.34.0^38^. For Alpha-diversity, pairwise comparisons between the groups were performed using a paired Wilcoxon rank sum test with the R package “Stats”^39^. Differential abundance analysis was performed using Songbird v1.0.4^40^. In short, Songbird is a compositionally aware differential abundance method which provides rankings of features based on their log fold change with respect to covariates of interest. It uses a Differential Ranking (DR) method reformulating the differential abundance analysis as a multinomial regression problem. In this case, we used the formula and parameters “songbird multinomial –formula "C (Group, Treatment ('nis_T72'))" –epochs 10,000—differential-prior 0.5 –summary-interval 1 –min-sample-count 1” where “nis_T72” corresponds to the groups Nis-en T72 and Nis-pdr T72.

We selected the 20 highest and 20 lowest ranked Metaphlan3 species associated with Nis-en and Nis-pdr at T72 and computed the log ratio of these sets of taxa. Comparing the ratios of taxa in this way mitigates bias from the unknown total microbial load in each sample and taking the log of this ratio gives equal weight to relative increases and decreases of taxa. Evaluation of the Songbird model for Nis-en and Nis-pdr at T72 against a baseline model obtained a Q2 value of 0.323.

### Functional composition and group comparisons

Genes associated with SCFAs were extracted from the go_uniref90gene table both stratified by species and unstratified, normalised to count per million (cpm). The following genes were used for the different SCFAs: acetate kinase (ackA) for acetate, butyrate kinase (buk) and butyryl-CoA: acetate CoA transferase (but) for butyrate and methylmalonyl-CoA decarboxylase (mmdA), lactoyl-CoA dehydratase (lcdA), and CoA-dependent propionaldehyde dehydrogenase (pduP) for propionate. Reads from different genes were added for each SCFA to give the representative value which were used in the analysis. Log2 fold changes were calculated for each group related to BL. Differential abundance analysis of HUMAnN pathways (both stratified and unstratified) was also performed with Songbird. We used the same formula and parameters as for the taxonomic differential abundance analysis described previously. Evaluation of the Songbird model for Nis-en and Nis-pdr at T72 against a baseline model obtained a Q2 value of 0.154.

### Statistical analysis and data visualisation

#### Two-group univariate comparisons

Statistical significance was calculated using Wilcoxon rank-sum test (using the function “wilcox.test” as implemented in R 4.0.2), with paired option. The p-values were adjusted for false discovery using Benjamini–Hochberg procedure.

#### Multivariate statistical analyses

Principal Coordinate Analysis (PCoA) were conducted based on the relative abundance of bacterial species and HUMAnN gene counts (counts per million) in each faecal metagenome as assessed using Bray–Curtis distances. Significance of the clustering by variables (i.e., nisin treatment vs control groups) was determined by Permutational Multivariate Analysis of Variance (PERMANOVA) with 1000 permutations (R packages “PhyloSeq”, “vegan”, and “pairwiseAdonis”).

R version 4.0.2 was used for the statistical analysis and visualisation of the results.

### Ethical approval and consent to participate

This study was approved by the Teagasc Animal Ethics Committee (TAEC185-2018) and an experimental licence number (AE19132/P083) was obtained from the Irish Health Products Regulatory Authority (HPRA). All procedures were performed according to the European Union Directive 2010/63/EU on the protection of animals for scientific purposes. The study is reported in accordance with ARRIVE guidelines (https://arriveguidelines.org).

## Supplementary Information

Supplementary Information.

## Data Availability

Codes used for bioinformatics and statistical analysis will be deposited on Github when the manuscript will be accepted for publication. Meanwhile, codes can be shared upon request. Shotgun metagenomics data generated and/or analysed during the current study are available in the on NCBI under the project name PRJNA906489 (https://www.ncbi.nlm.nih.gov/sra/PRJNA906489).
