# Supplementary material for: Modulation of the gut microbiome with nisin
Source: Sci Rep. 2023 May 16;13:7899. doi: 10.1038/s41598-023-34586-x (PMC10188554; doi:10.1038/s41598-023-34586-x)
Supplement: Supplementary file 1 — Supplementary Information. [file 41598_2023_34586_MOESM1_ESM.docx]

**Supplementary Information**

**Supplementary Table 1**: Proximate analysis, chemical composition and amino acid composition of the basal diets used in this study (as-fed basis).

| **Analysis:** | **Unitsa** | **Starter Feed** | **Link Feed** |
| --- | --- | --- | --- |
| **Dry Matter** | MJ/kg | 18.4 | 17.7 |
| **Chemical Analysis:** |  |  |  |
| Neutral Detergent Fibre | % | 4.8 | 5.5 |
| Crude Protein (N x 6.25) Dumas | % | 19.6 | 18.9 |
| Crude Fibre | % | 1.6 | 2.3 |
| Dry Matter | % | 91.6 | 90.3 |
| Ash | % | 5.8 | 5.3 |
| Total Oil (Oil B) | % | 11.8 | 8.0 |
| **Amino Acids Composition*:** |  |  |  |
| Alanine | % | 0.72 | 0.71 |
| Arginine | % | 1.06 | 1.08 |
| Aspartic | % | 1.77 | 1.70 |
| Cysteine | % | 0.22 | 0.25 |
| Glutamic | % | 3.28 | 3.31 |
| Glycine | % | 0.63 | 0.66 |
| Histidine | % | 0.47 | 0.45 |
| Iso-Leucine | % | 0.81 | 0.75 |
| Leucine | % | 1.49 | 1.38 |
| Lysine | % | 1.56 | 1.43 |
| Methionine | % | 0.63 | 0.53 |
| Phenylalanine | % | 0.88 | 0.83 |
| Proline | % | 1.10 | 1.08 |
| Serine | % | 0.91 | 0.85 |
| Threonine | % | 1.01 | 0.96 |
| Tyrosine | % | 0.41 | 0.33 |
| Valine | % | 0.99 | 0.94 |
| Tryptophan | % | 0.32 | 0.29 |

^a^MJ per kilogram and percentage (%); *all amino acids are expressed as total e.g. alanine (total).

**Supplementary Table 2:** Levels of SCFA (mM) detected across treatment groups and time points (average over samples).

| **Time point** | **Treatment** | **Acetate** | **Isobutyric acid** | **Isovaleric acid** | **Propionic acid** | **Butyrate** | **Valeric acid** |
| --- | --- | --- | --- | --- | --- | --- | --- |
| **mM (average over samples)** | | | | | | | |
| Baseline | Control | 19.280 | 0.939 | 0.605 | 7.644 | 3.731 | 1.018 |
|  | Encapsulant | 13.756 | 0.685 | 0.486 | 5.841 | 2.871 | 0.658 |
|  | Encapsulated Nisin | 14.137 | 0.721 | 0.473 | 5.272 | 2.685 | 0.624 |
|  | Nisin Powder | 16.763 | 0.954 | 0.646 | 6.766 | 3.298 | 1.002 |
| 72 h after fed initial treatment | Control | 23.257 | 1.260 | 0.903 | 10.130 | 6.089 | 1.637 |
|  | Encapsulant | 18.708 | 1.054 | 0.748 | 7.924 | 5.798 | 1.467 |
|  | Encapsulated Nisin | 16.134 | 0.852 | 0.588 | 8.836 | 4.166 | 1.373 |
|  | Nisin Powder | 13.816 | 0.840 | 0.609 | 8.515 | 2.987 | 1.266 |
| 10 days after treatment stopped | Control | 23.025 | 1.080 | 0.769 | 11.174 | 7.183 | 1.750 |
|  | Encapsulant | 20.627 | 0.999 | 0.711 | 9.741 | 6.355 | 1.734 |
|  | Encapsulated Nisin | 19.806 | 0.874 | 0.625 | 9.369 | 5.023 | 1.240 |
|  | Nisin Powder | 16.383 | 0.893 | 0.659 | 7.140 | 4.493 | 1.176 |

(a)


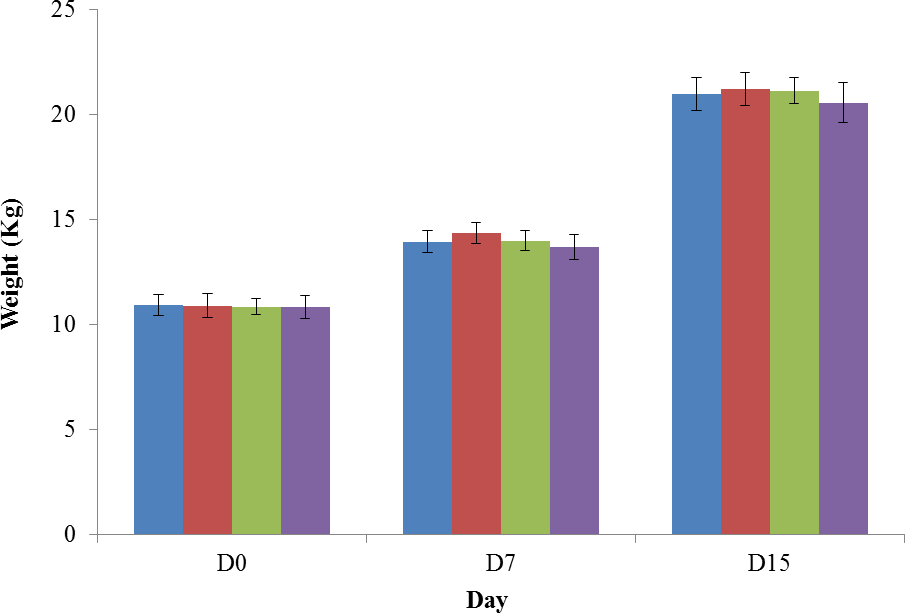


(b)


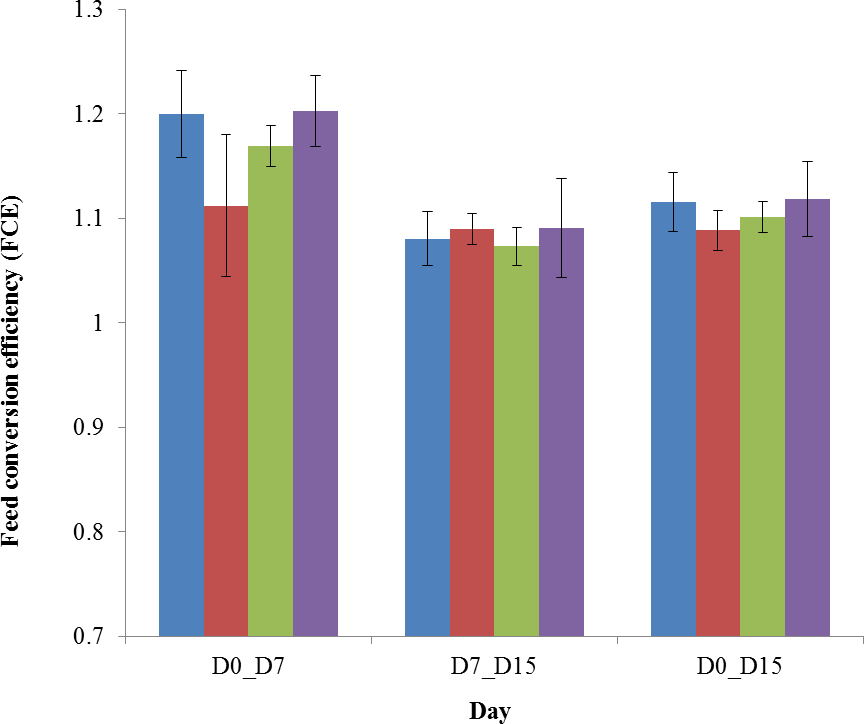


**Supplementary Figure 1.** Mean live weight (a) and feed conversion efficiency (FCE) (b) of post-weaning piglets by across the days of weighing over the trial period. Treatments; ◼ Ctl,

- Encap, ◼ Nis-pdr and ◼ Nis-en.

Relative abundance (%)

**Supplementary Figure 2.** Relative abundance (%) at the genus level across time points and treatment groups. The four treatment diets are shown here: no treatment control (Ctl); encapsulant material (Encap); encapsulated nisin (Nis-en) and nisin powder (Nis-pdr). Time points: Baseline (BL), 24 h after initial treatment fed (T24), 48 h after initial treatment fed (T48), 72 h after initial treatment fed (T72), 3 days after feeding treatments ceased (3d PT) and 10 days after feeding treatments ceased (10d PT).


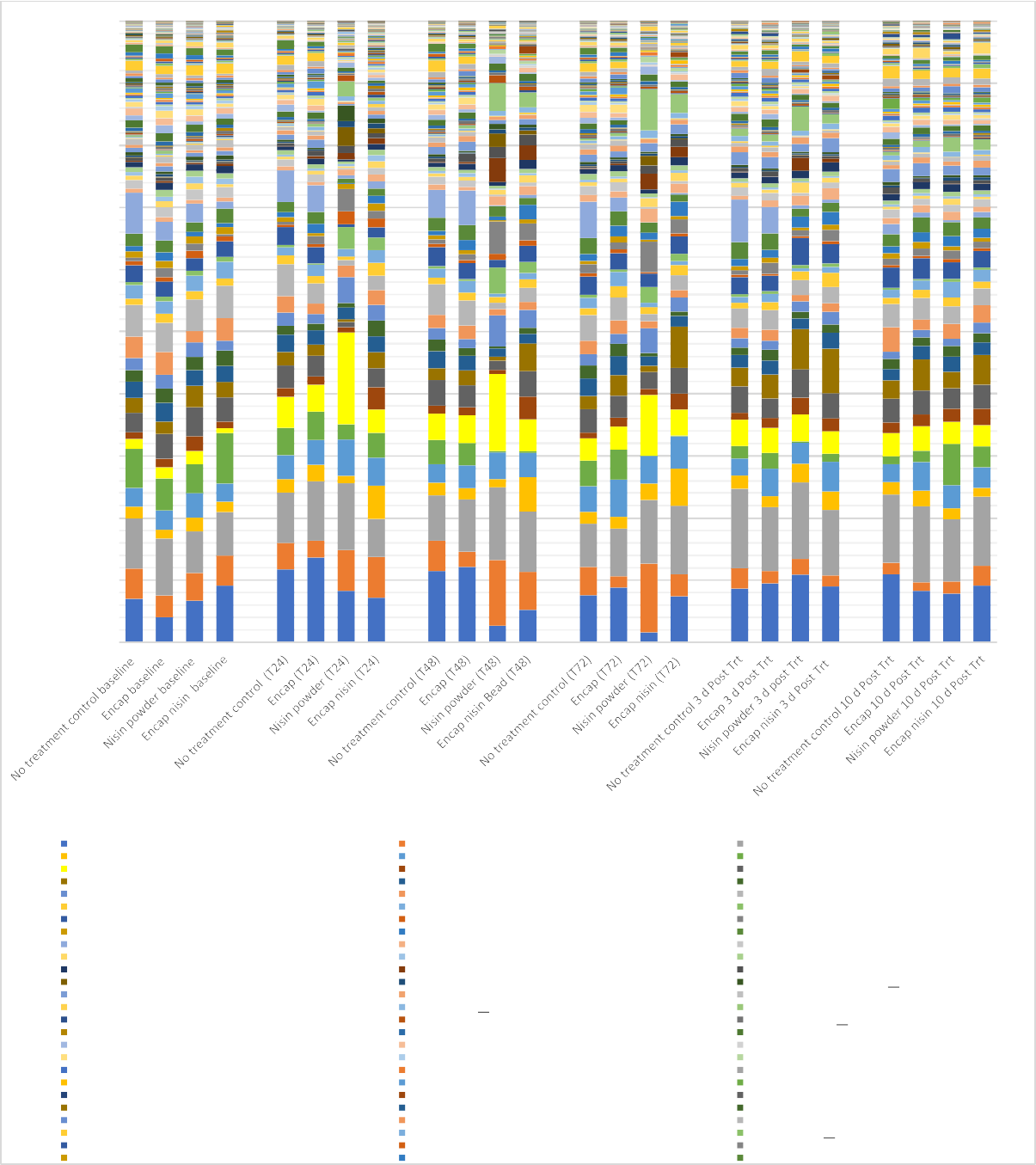


100%

90%

80%

70%

60%

50%

40%

30%

20%

10%

0%

Treatment by Day

Lactobacillus

Faecalibacterium Megasphaera Catenibacterium Phascolarctobacterium Ruminococcaceae.UCG.002 Blautia Ruminococcaceae.UCG.010 Streptococcus Holdemanella Ruminococcus.1

Klebsiella Roseburia

Lachnospiraceae.NK4A136.group Ruminiclostridium.6 Lachnospiraceae.XPB1014.group Unassigned.Other Solobacterium

Oribacterium Gastranaerophilales.uncultured.bacterium. Ruminococcaceae.UCG.013 Candidatus.Soleaferrea Ruminococcaceae.UCG.014

Prevotella 2 Oscillibacter Asteroleplasma

Prevotella 1

BacteroidalesS247groupunculturedbacterium Prevotella 7

Rikenellaceae.RC9.gut.group Treponema.2 Peptoclostridium Erysipelotrichaceae.UCG.004 Subdoligranulum

.Eubacterium..coprostanoligenes.group Campylobacter

EscherichiaShigella Fibrobacter Lachnoclostridium Veillonellaceae uncultured Sphaerochaeta Family.XIII.AD3011.group

.Eubacterium..hallii.group Methanobrevibacter Desulfovibrio Clostridium.sensu.stricto.6 Fusicatenibacter Lachnospiraceae.FCS020.group Marvinbryantia

Helicobacter Candidatus.Saccharimonas Collinsella

Prevotella 9

Clostridium.sensu.stricto.1 FLachnospiraceae.Other Christensenellaceae.R.7.group uncultured

Parabacteroides Succinivibrio

Prevotellaceae.NK3B31.group Ruminococcaceae.UCG.005 Ruminococcaceae.NK4A214.group Anaerovibrio

Clostridiales.vadinBB60.group. uncultured.bacterium Lachnospiraceae.ND3007.group

Mitsuokella

Planctomycetaceae p.1088.a5.gut.group Alloprevotella BacteroidalesRF16groupunculturedbacterium Clostridiales uncultured

Butyricicoccus Anaerostipes

Clostridiales.vadinBB60.group.Other Coprococcus.1 Prevotellaceaeuncultured

Bradymonadales uncultured.bacterium Lachnospiraceae.UCG.004 Prevotellaceae.UCG.003

UBISYN-PWY: superpathway of ubiquinol-8 biosynthesis (prokaryotic)­

PWY3O-355: stearate biosynthesis Ill (fungi)­ PWY0-881: super-pathway of fattyacidbiosynthesis I (E. coli) -

PWY- 7446: sulfogtycolysis - PWY-7345: superpathway of anaerobic sucrose deg:radation-

PWY- 7269: NAO/NAOP-NAOH/NAOPH mitochondrial interconversion (yeast) -

PWY-6708: ubiqu.inol-8 OOsynthesis (prokaryotic)- PWY--6285: superpathway of fatty acids biosynthesi.s (E. coli) - PWY-5899: supe-rpathway of menaquinol-13 l)josynthesis - PWY-5898: supe-rpathway of menaquinol-12 biosynthesis - PWY-5897: supe-rpathway of menaquinol-11 biosynthesis - PWY-5896: supe-rpathway of menaquinol-10 biosynthesis -

PWY-5862: superpathway of demethylmenaquinol-9 biosynthesis­ PWY-5861: superpathway of demethylmenaquinol-8 biosynthesis - PWY-5860: supe-rpathway of demethylmenaquinol-6 biosynthesis I - PWY-5857: ubiquinol- tO biosynthesis (prokaryotic) -

PWY-5856: ubiquinol-9 biosynthesis (prokaryotic)­ PWY-5855: ubiquinol- 7 biosynthesis (prokaryotic) -

PWY-5850: supe-rpathway of menaquinol-6 biosynthesis I- PWY-5845: superpathway of menaquinol-9 biosynthesis­ PWY-5840: superpathway of menaquinol- 7 biosynthesis - PWY-5838: supe-rpathway of menaquinol-8 biosynthesis 1- PWY-5837: 1.4-dihydroxy-2-naphthoate biosynthesis I­

PWY-5791: 1,4-dihydroxy-2-naphthoate biosynthesis II {plants)­ PWY-5656: mannosylglycerate biosynthesis I­

PWY-3801: sucrosedegradation II (sucrose synthase) -

P221-PWY: octane oxidation- P162-PWY: L-glutamate degradation V (via hydroxyglutarate) - LPSSYN-PWY: superpathway of li:popolysaocharide biosynthesis - ENTBACSYN-PWY: ente-robactin biosynthesis­

ECASYN-PWY: enterobacterial common antigen biosynthesis- 3-HYOROXYPHENYLACETATE-DEGRAOATION-PWY: 4-hydroxyphenyiacetate degradation -

Ctl Encap Nis-en Nis-pdr


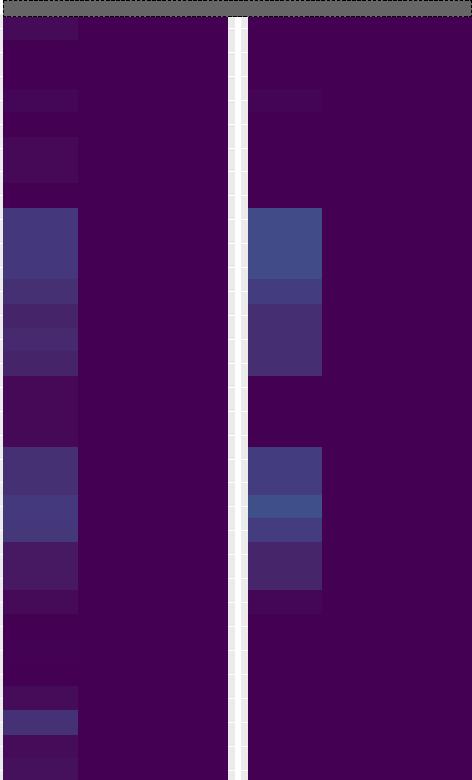
r ,


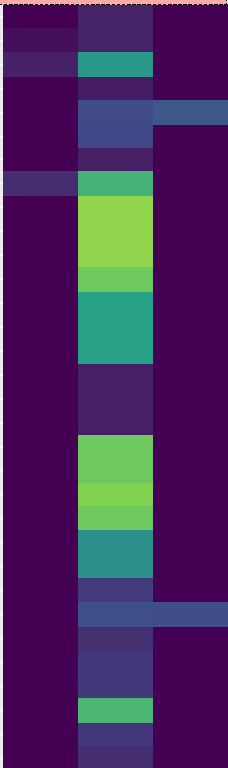


'


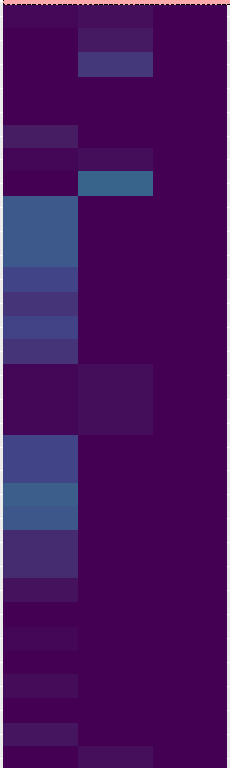
'

Basefine T72 IOd PT Baseline T72 10dPT Baseline T72 10d PT Baseline T72 10d PT

CPM

#
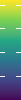
100

75

# 50

25

0

**Supplementary Figure 3_** Heatmap showing the abundance (HUMAnN count per million) of the most differentially abundant HUMAnN pathways (low level hierarchy, unstTatified) in nisin at baseline vs nisin tTeatment at T72 for all the different groups at the different time points (grey area, conti-ol groups, pink area, tTeatment groups), as found according to the Songbird analysis (model: pathways~ nisin tTeatment x days)


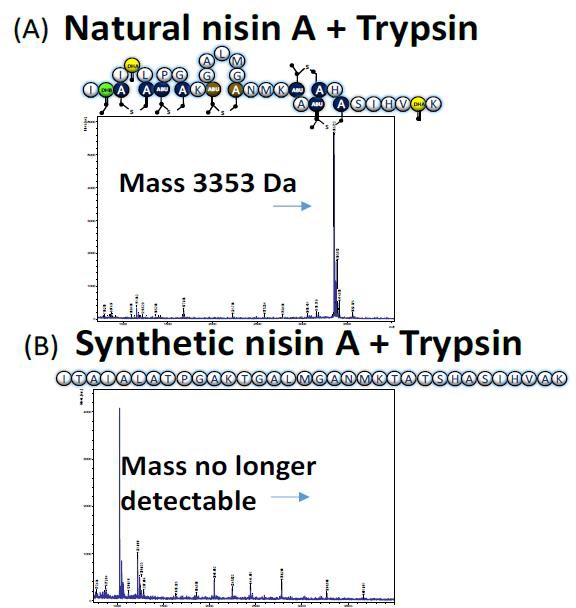


**Supplementary Figure 4** (A) Post translationally modified natural nisin treated with trypsin. Nisin mass detected by MS (B) Synthetic nisin with no post-translational modifications treated with trypsin. No nisin mass detected by MS


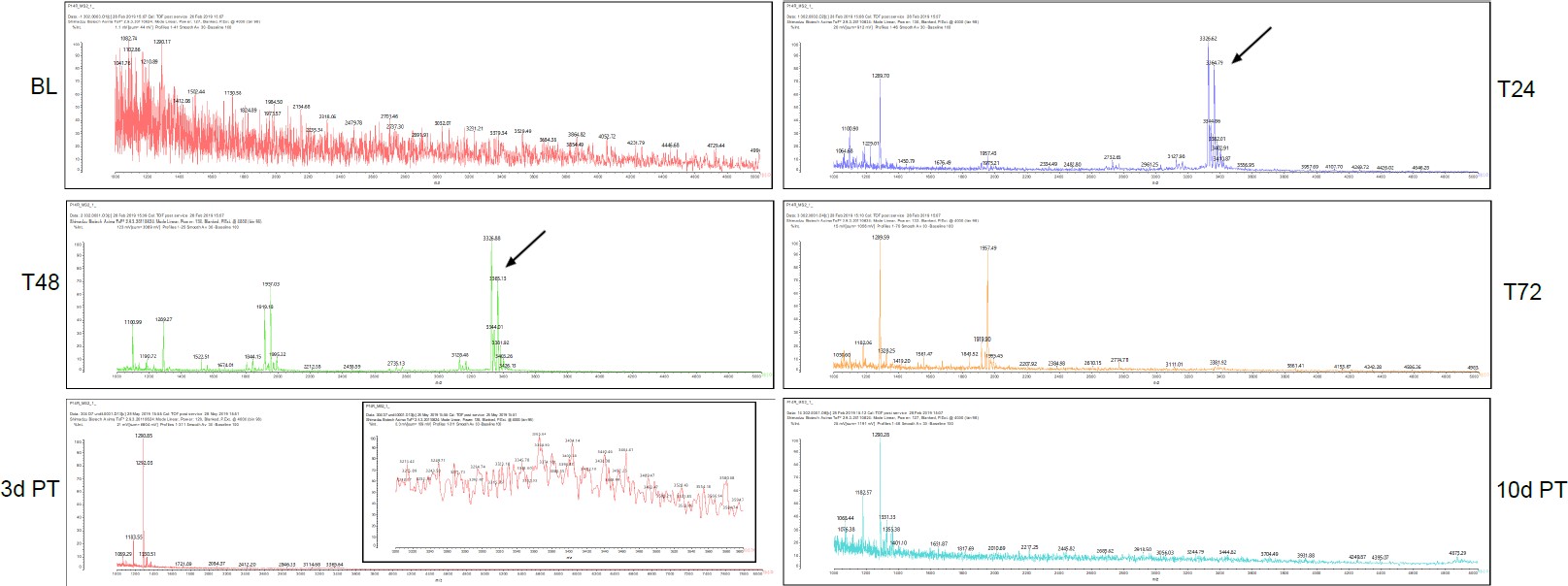


**Supplementary Figure 5** MALDI TOF Mass spectrophotometry analysis to detect nisin (black arrows denote intact nisin peaks). Detection of intact nisin in the faeces of pig in the nis-en treated group at Baseline (BL), 24 hr following initial treatment (T24), 48 hr following initial treatment (T48), 72 hr following initial treatment (T72), 72 hr after initial treatment stopped (3d PT) and 10 days after initial treatment stopped (10d PT). Smaller zones and Nisin masses were found in T72 and no zones or masses found on 3d PT and 10d PT (1 in 10 dil).
